# Carboxypeptidase E Modulates Intestinal Immune Homeostasis and Protects against Experimental Colitis in Mice

**DOI:** 10.1371/journal.pone.0102347

**Published:** 2014-07-22

**Authors:** Florian Bär, Bandik Föh, René Pagel, Torsten Schröder, Heidi Schlichting, Misa Hirose, Susanne Lemcke, Antje Klinger, Peter König, Christian M. Karsten, Jürgen Büning, Hendrik Lehnert, Klaus Fellermann, Saleh M. Ibrahim, Christian Sina

**Affiliations:** 1 Medical Department I, University Hospital Schleswig-Holstein, Lübeck, Germany; 2 Department of Dermatology, University Hospital Schleswig-Holstein, Lübeck, Germany; 3 Institute of Anatomy, University of Lübeck, Lübeck, Germany; 4 Institute for Systemic Inflammation Research, University of Lübeck, Lübeck, Germany; Duke University Medical Center, United States of America

## Abstract

Enteroendocrine cells (EEC) produce neuropeptides, which are crucially involved in the maintenance of the intestinal barrier. Hence, EEC dysfunction is suggested to be involved in the complex pathophysiology of inflammatory bowel disease (IBD), which is characterized by decreased intestinal barrier function. However, the underlying mechanisms for EEC dysfunction are not clear and suitable models for a better understanding are lacking. Here, we demonstrate that Carboxypeptidase E (CPE) is specifically expressed in EEC of the murine colon and ileum and that its deficiency is associated with reduced intestinal levels of Neuropeptide Y (NPY) and Peptide YY (PYY), which are both produced by EEC. Moreover, *cpe^−/−^* mice exhibit an aggravated course of DSS-induced chronic colitis compared to wildtype littermates. In addition, we observed elevated mucosal IL-6 and KC transcript levels already at baseline conditions in *cpe^−/−^* mice. Moreover, supernatants obtained from isolated intestinal crypts of *cpe^−/−^* mice lead to increased IL-6 and KC expression in MODE-K cells in the presence of LPS. This effect was reversible by co-administration of recombinant NPY, suggesting a CPE mediated immunosuppressive effect in the intestines by influencing the processing of specific neuropeptides. In this context, the chemotaxis of bone marrow derived macrophages towards respective supernatants was enhanced. In conclusion, our data point to an anti-inflammatory role of CPE in the intestine by influencing local cytokine levels and thus regulating the migration of myeloid immune cells into the mucosa. These findings highlight the importance of EEC for intestinal homeostasis and propose EEC as potential therapeutic targets in IBD.

## Introduction

Inflammatory bowel diseases (IBD) are chronically recurring inflammatory disorders of the gastrointestinal tract (GIT) characterized by an impaired intestinal barrier function [1]. Although extensive research has been performed over the last decades, the underlying mechanisms are still unknown.

According to current knowledge, the maintenance of the intestinal barrier critically depends on the interplay of the gut microbiota, intestinal epithelial cells (IEC) and primary immune cells [2], [3]. The orchestration of these cells is tightly organized and depends on various messenger molecules such as cytokines and neuropeptides [3]–[5]. Consequently, a disturbance in this communication network is suggested to contribute to the pathogenesis of IBD [6].

In IBD patients, intestinal levels of neuropeptides expressed by enteroendocrine cells (EEC) are altered [7]–[10], leading to the hypothesis of EEC dysfunction as an underlying pathophysiological mechanism [11]. Supporting this theory is the association between Crohn's Disease (CD) and autoantibodies against the ubiquitination protein 4a (UbE4A), a protein with specifically high expression in EEC [12]. Additionally, gene polymorphisms in the EEC transcription factor Phox2B are present in CD, which might affect expression levels of EEC derived neuropeptides in CD [11], [13].

However, animal models employed to determine the role of neuropeptides in intestinal inflammation focused only on single neuropeptides and therefore disregarded the complex interplay of different neuropeptides for intestinal homeostasis. Considering that EEC function is exerted by a great number of different neuropeptides, modulators of neuropeptide synthesis turn up as potential tools to examine the influence of EEC on intestinal inflammation.

CPE is an exopeptidase crucial for the function of neuroendocrine cells by processing and sorting of many neuropeptides. Moreover, there is increasing evidence for a CPE expression in EEC [14] implicating a functional role for EEC function and therefore for intestinal homeostasis and barrier function.

In this study we were able to show the specific expression of CPE in EEC of the lower intestine by immunofluorescence staining. After we confirmed a relevant role of CPE on neuropeptide levels in the GIT, we used *cpe* ^−/−^ mice as a model of EEC dysfunction to examine the pathophysiologic mechanisms in experimental colitis. As a result we provide evidence for a relevant role of CPE for EEC function and intestinal homeostasis. As a new model for EEC dysfunction *cpe* ^−/−^ mice might contribute to new insights in the pathophysiology of IBD.

## Materials and Methods

### Ethics Statement

All mice were placed in the Laboratory Animal Unit of the University of Lübeck, kept in a climate-controlled environment with 12 hour light-dark-cycle and were provided with food and water ad libitum. All experiments were performed in strict accordance with the animal care guidelines of the University of Lübeck and with national and international laws and policies. The study protocol was approved by the Ministerium für Energiewende, Landwirtschaft, Umwelt und ländliche Räume des Landes Schleswig-Holstein (acceptance no.: V 312–72241.122-1 (70–6/11)). During the experiments all efforts were made to minimize suffering.

### Animal models

All experiments were performed in accordance with the animal care guidelines of the University of Lübeck (acceptance no.: V 312–72241.122-1 (70–6/11)). Procedures involving animals and their care were conducted in accordance with national and international laws and policies. *Cpe* ^+/+^ and *cpe* ^−/−^ mice were generated by crossing B6.HRS(BKS)-*Cpe^fat^*/J^+/−^ mice obtained from the Jackson Laboratory. In all experiments *cpe* ^−/−^ mice and their respective littermate controls (*cpe* ^+/+^) were used. The animals were kept under standard conditions at the animal facility of the University to Lübeck and were provided with food and water ad libitum.

### Immunofluorescence analysis of mouse intestine

Tissue slides of ileal and colonic biopsies were immunostained with anti-Chromogranin B (CgB), anti-CPE, anti-NPY, anti-CD3, anti-F4/80 and anti-Ly6G and anti-Peptide YY (PYY) according to standard protocols. Briefly, frozen tissue sections were fixed in methanol and acetone (1∶1) and then incubated with goat anti-CgB (Santa Cruz, 1∶100), rabbit anti-CPE (Sigma, 1∶200), goat anti-NPY (Santa Cruz, 1∶200), goat anti-CD3 (BioLegend, 1∶1000), goat anti-F4/80 (BioLegend, 1∶10000), or goat anti-PYY (Santa Cruz, 1∶200) primary antibodies for one hour. After several washing steps with PBS sections were incubated with appropriate secondary antibodies. Nuclear staining was done with Hoechst dye, and slides were evaluated using a confocal laser scanning microscope.

### Induction of Colitis and determination of clinical scores

In two independent experiments, chronic colitis induction was performed in *cpe* ^−/−^ mice and their littermates (*cpe* ^+/+^) at an age of 9–10 weeks by feeding with 2% dextran sulfate sodium (DSS; molecular mass 40 kDa; TdB Consultancy; Uppsala; Batch DB001-27) dissolved in the drinking water for 5 days, followed by 5 days of normal drinking water. This cycle was repeated three times. Control mice received water without DSS. Mice were sacrificed on day 30 of the experiment as described previously [15]. Clinical parameters were assessed every other day. The disease activity index (DAI) was assessed as a combined score of weight loss, stool consistency and rectal bleeding as described elsewhere [16]. High resolution mouse endoscopy was employed (HOPKINS Optik 64019BA; Karl Storz AidaVet) to determine the murine endoscopic index of colitis severity (MEICS) as described previously [15], [17].

### Histological analysis of mouse colon tissue

Ileal and colonic biopsies were fixed in 4% formalin and embedded in paraffin. Sections of 5 µm thickness were stained with haematoxylin-eosin using standard procedures. The grade of inflammation after chronic DSS-colitis induction was determined as described elsewhere [16]. Briefly, the grade and extent of tissue damage was assessed and the infiltration with inflammatory cells was estimated. The analysis was conducted by two independent observers in a blinded fashion.

### Real time PCR

RNA was extracted using the innuPREP RNA mini kit (Analytik Jena AG, Germany) and transcribed to cDNA (RevertAid H Minus reverse transcriptase, Fermentas). Real-time quantitative PCR was carried out with 10 µl of Maxima SYBR Green qPCR Master Mix, plus 0.5 µM of each primer using a 96-well plate format. The amplification program consisted of: i) pre-incubation at 95°C for 5 min; ii) 40 cycles of denaturation at 95°C for 45 s and annealing at appropriate temperature (60°C) for 1 min using ABI PRISM 7000 (Applied Biosystems). To confirm amplification of specific transcripts, melting curve profiles were produced. Expression levels were normalized to β-Actin. The following primer pairs were used: β-Actin (for: GATGCTCCCCGGGCTGTATT, rev: GGGGTACTTCAGGGTCAGGA), IL-6 (for: CTCCCAACAGACCTGTCTATAC, rev: GTGCATCATCGTTGTTCATAC), KC (for: GCTGGGATTCACCTCAAGAA, rev: TGGGGACACCTTTTAGCATC), TNF-α (for: CCCTCACACTCAGATCATCTTCTC, rev: TGGCTCAGCCACTCCAG), CgB (for: CCCGCTGGCTGAACTTTTC, rev: GAGTTCTGACGGCGGAAGAG), PYY (for: TTCACAGACGACAGCGACA, rev: CACCACTGGTCCAAACCTTC), NPY (for: CCCTCGCTCTATCTCTGCTCGT, rev: GCGTTTTCTGTGCTTTCCTTCA), VIP (for: TGGATGACAGGATGCCGTTT, rev: CGGCATCAGAGTGTCGTTTG), CgA (for: AGCCAGACTACAGACCCACT, rev: TGACTTCCAGGACGCACTTC).

### ELISA

Colonic punch biopsies were obtained from both genotypes and stimulated with lipopolysaccharide (LPS) (50 ng/ml) or forskolin (30 µM), an agent with previously described secretagogue effects [18], for two hours at 37°C. Culture supernatants were then harvested and assayed for NPY and PYY using commercially available kits according to the manufacturer's instructions (Mouse NPY ELISA KIT: Bio-Medical Assay Co.,Ltd. Cat#: 21176, Mouse PYY EIA KIT: Ray Biotech, Inc. Cat#. EIA-PYY-1).

### Cell culture

Colonic crypts of both genotypes were isolated, treated with forskolin (30 µM) for one hour and supernatants were then added to an immortalized epithelial cell line derived from the murine duodenum (MODE-K) [19]. After 2 h of cultivation w/wo LPS (50 ng/ml) at 37°C and 5% CO_2_ cells were harvested for RNA preparation. In a subgroup of experiments recombinant NPY and PYY (Tocris Bioscience, Bristol, UK) were co-administered with LPS at a concentration of 1 µM.

### Migration assay

Femurs and tibias from wildtype mice were harvested and flushed with DMEM-medium containing 1% L-glutamin, 1% antibiotics and 10% fetal calf serum. Bone marrow cells were collected and differentiated to bone marrow-derived macrophages (BMDM) in DMEM-Medium containing 33% L929-cell culture supernatants as a source of M-CSF for 8 days at 37°C and 5% CO_2_. The chemotactic effects of crypt supernatants, which were harvested from *cpe* ^+/+^ and *cpe* ^−/−^ mice and pre-incubated with forskolin (30 µM) for two hours at 37°C, on bone marrow-derived macrophages were investigated as described elsewhere [20]. BMDM were resuspended in the same DMEM medium at a density of 3x10^6^ cells/ml. The crypt supernatants were used as chemoattractants, placed in the bottom wells of a micro Boyden chemotaxis chamber (Neuroprobe) and overlaid with a 5 µm polycarbonate membrane. 50 µl of the BMDM cell suspension were then placed in the top wells and incubated for 45 min at 37°C and 10% CO_2_. Subsequently, the membranes were removed and the cells on the bottom side of the membrane were stained with Diff-Quick. The numbers of migrated cells per high-power field were counted by light microscopy. Results are expressed as percentage of the mean value of wildtype mice. The Assay was replicated twice independently.

### Statistical analysis

Results are expressed as means ± SEM. Values of *p* were calculated with Prism 5 (GraphPad) using a 2-tailed Student's t test for parametric values and MANN-Whitney-U test for non-parametrical values. Values of *p*<0.05 were considered statistically significant. Experiments and measurements were replicated at least twice.

## Results

### CPE co-localizes with CgB, NPY and PYY in the lower murine intestine

**Figure 1:**
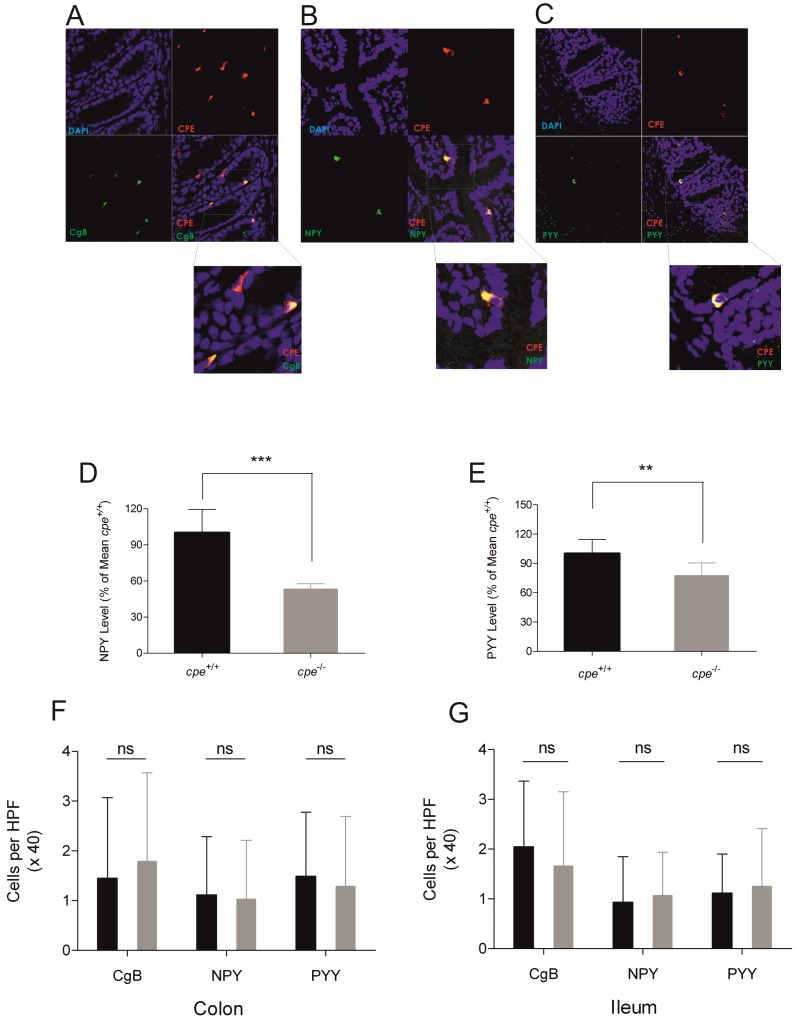
CPE is expressed in EEC and affects colonic NPY and PYY levels. (A–C) Immunofluorescence staining of colonic biopsies from wildtype mice for CPE and the EEC marker CgB (A), NPY (B) and PYY (C). (D–E) NPY (D) and PYY (E) neuropeptide level assayed by ELISA from LPS treated colon punch biopsies. n = 8 per genotype. (F–G) Determination of enteroendocrine cell numbers in the colonic (F) and ileal (G) mucosa by counting CgB, NPY and PYY positive epithelial cells per high power field (magnification 40x), 5 random HPF per animal, n = 6 per genotype. **p<0.01, ***p<0.001, ns =  not significant, by t-test.

Although CPE has been detected in enteroendocrine cells of the upper GIT [14], nothing is known about CPE expression in the lower intestines. Therefore, we performed a CPE-specific staining of intestinal biopsies showing that CPE is located in defined cells of the ileal and colonic epithelium. Furthermore, staining for NPY, which is known to be processed by CPE [21]–[23], as well as CgB and PYY revealed a co-localization of these EEC markers with CPE (Figure 1A, B, C; ileum not shown). In conclusion, our results demonstrate a specific expression of CPE in enteroendocrine cells.

### CPE deficiency causes reduced colonic levels of NPY and PY*Y*

In order to investigate the effects of CPE deficiency on EEC function, we next determined the levels of EEC-secreted neuropeptides NPY and PYY in supernatants of colonic biopsies from *cpe* ^−/−^ mice and wildtype controls. We did not detect relevant differences of neuropeptides in colonic supernatants at baseline (data not shown). Therefore, we examined the levels of neuropeptides after addition of LPS, a known inducer of neuropeptide secretion which is constantly present in the gut under physiological conditions [24], [25]. NPY and PYY level were found to be significantly decreased in *cpe* ^−/−^ mice compared to wildtype controls (Figure 1D, E) after LPS treatment (NPY: 53.64%±3.99 (*cpe* ^−/−^) vs. 100%±19.33 (*cpe* ^+/+^), p<0.005; PYY: 77,98%±12.53 (*cpe* ^−/−^) vs. 100%±20.04 (*cpe* ^+/+^), p<0,01). However, transcript levels of NPY and PYY as well as other neuropeptides such as Chromogranin A (CgA), Chromogranin B and vasoactive intestinal peptide (VIP) were slightly increased in *cpe* ^−/−^ mice, although not reaching statistical significance (Figure S1A). This could indicate a mechanism to compensate the reduced levels of neuropeptides due to CPE-deficiency.

In order to check if the decreased neuropeptide levels in *cpe* ^−/−^ mice are due to a reduced quantity of enteroendocrine cells, we further counted CgB, NPY and PYY positive cells as cell specific markers for EEC. No significant differences in enteroendocrine cell counts could be observed in ileal and colonic biopsies (Figure 1F, G), indicating that indeed CPE deficiency is causative for the neuropeptide reduction.

### CPE deficiency aggravates experimental chronic colitis

**Figure 2:**
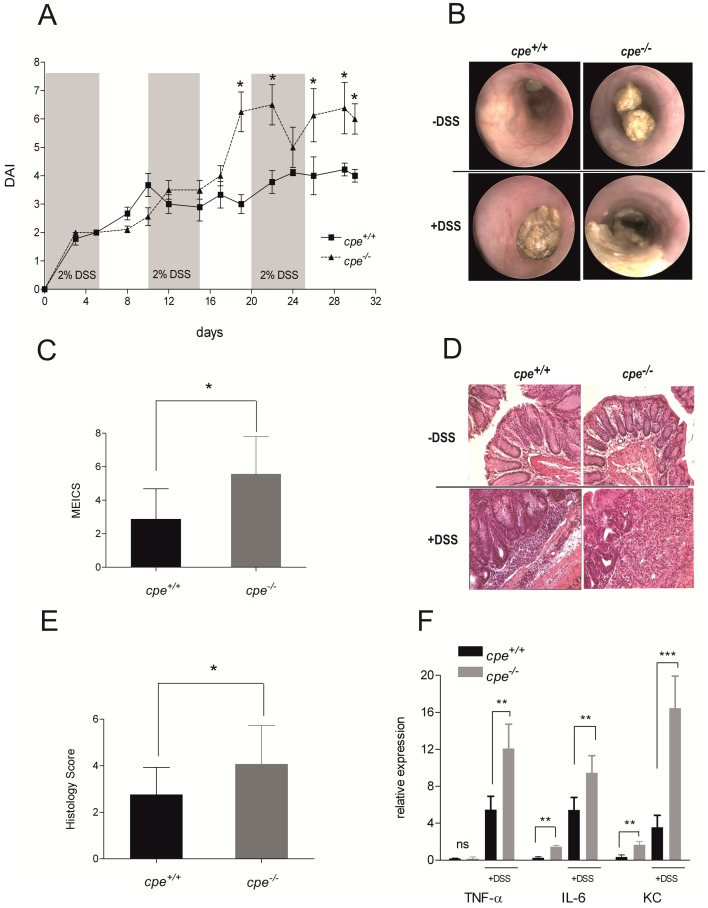
CPE deficiency aggravates experimental chronic colitis. (A) Calculation of the disease activity index (DAI) by determining clinical parameters of inflammation (body weight development, stool consistency, rectal bleeding) through 30 days of experimental colitis. n = 9 (*cpe* ^+/+^), n = 8 (*cpe* ^−/−^). (B-C) Determination of macroscopic colitis severity via mouse endoscopy. Representative endoluminal pictures of the distal colon on day 30 of experimental colitis (B) and calculation of the murine endoscopic index of colitis severity (MEICS) by analyzing mucosal morphology, stool consistency and shape of the vascular pattern via mouse endoscopy (C). n = 9 (*cpe* ^+/+^), n = 8 (*cpe* ^−/−^). (D–E) Determination of microscopic colitis severity via histology. Representative histological pictures of the distal colon on day 30 of experimental colitis (D) and calculation of the histology score by analyzing mucosal architecture and infiltration of immune cells (E). n = 8 per genotype. (F) Determination of expression level of TNF-α, IL-6 and KC in colonic punch biopsies by real time RT-PCR after 30 days of experimental colitis and at baseline. n = 8 per genotype. *p<0.05, **p<0.01, ***p<0.001 by t-test.

Considering a crucial role of EEC for intestinal barrier function, a feature that is highly disturbed in IBD [2], we next examined the effects of CPE deficiency on intestinal inflammation. Experimental chronic colitis was induced by cyclic administration of 2% DSS, and clinical parameters were evaluated. Regarding body weight loss, rectal bleeding and stool consistency, *cpe* ^−/−^ mice displayed a significantly more severe phenotype of colitis compared to wildtype controls (Figure 2A). Macroscopic assessment of colitis severity via mouse endoscopy confirmed the physical findings with a corresponding higher MEICS-Score in *cpe* ^−/−^ mice (5.57±2.23 (*cpe* ^−/−^) vs. 2.88±1.81 (*cpe* ^+/+^), p<0.05) (Figure 2B, C). Additionally, colonic biopsies were examined histologically and scored for mucosa morphology and inflammatory cell infiltration. Compared to wildtype controls, *cpe* ^−/−^ mice displayed a higher histology score comprised from the degree of mucosal edema, abscesses and ulcer as well as the numbers of infiltrating immune cells (4.07±1.66 (*cpe* ^−/−^) vs. 2.77±1.17 (*cpe* ^+/+^), p<0.05) (Figure 2D, E). Interestingly, at baseline conditions no visible histological differences of the mucosal/submucosal architecture were detected. Considering the proliferative potential of primary immune cells on EEC and their products as recently demonstrated by Worthington *et al.*, we counted CD3 (89.64%±9.353 (*cpe* ^−/−^) vs. 100%±11.84 (*cpe* ^+/+^), n.s.) and F4/80 (107.6%±14.66 (*cpe* ^−/−^) vs. 100%±9.65 (*cpe* ^+/+^), n.s.) positive cells in intestinal biopsies in order to ensure that our strains do not exhibit relevant different myeloid cell compositions in the intestines (Figure S1B–E) [26]. In summary, CPE deficiency, although not leading to spontaneous intestinal inflammation, is associated with increased susceptibility to pro-inflammatory intestinal stimuli.

### Cytokine profile of CPE-deficient mice

**Figure 3:**
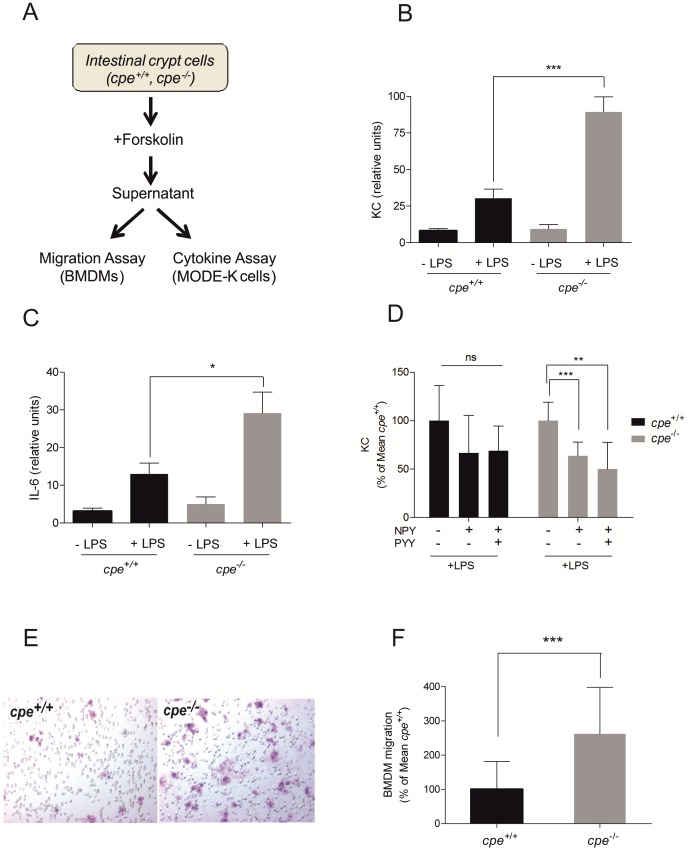
Proinflammatory properties of colonic crypt supernatants of CPE-deficient mice. (A) Experimental set-up for the acquirement and utilization of forskolin-stimulated supernatants of isolated colonic crypts. (B–C) Determination of KC (B) and IL-6 (C) transcript levels produced in MODE-K cells after incubation with LPS (50 ng/ml) and forskolin-stimulated supernatants of *cpe* ^+/+^ and *cpe* ^−/−^ colonic crypts via RT-PCR. (D). KC transcript levels produced in MODE-K cells after incubation with forskolin-stimulated supernatants of of *cpe* ^+/+^ and *cpe* ^−/−^ mice and LPS together with recombinant NPY +/− PYY (1 µM/ml). KC expression levels are expressed in percent of the Mean of *cpe^+/+^*. (E–F) BMDM migration via Boyden chamber assay. Representative pictures of migrated BMDM (E) and quantification (F) of BMDM migration towards supernatants of forskolin-stimulated colonic crypts of *cpe* ^+/+^ and *cpe* ^−/−^ mice. *p<0.05, **p<0.01, ***p<0.001 by t-test.

Cytokines are important factors in the communication of the local immune system in the gut and are tightly regulated by various factors, among them several neuropeptides [27]–[29]. Considering the decreased intestinal NPY and PYY levels in *cpe* ^−/−^ mice, we next analyzed colonic cytokine transcript levels of both genotypes at baseline conditions and after experimental colitis induction via quantitative RT-PCR. Under inflammatory conditions, *cpe* ^−/−^ mice showed significantly increased expression levels of tumor necrosis factor alpha (TNF-α), interleukin 6 (IL-6) and chemokine (C-X-C motif) ligand 1 (KC) compared to wildtype controls (Figure 2F), whereas levels of interferon gamma (IFN-γ), IL-5, IL-13 and IL-17 were not different (data not shown). Surprisingly, IL-6 and KC transcripts in *cpe* ^−/−^ mice were already significantly elevated at baseline (Figure 2F). Moreover, as IL-6 and KC are known to be expressed by IEC [30]–[32], we asked whether the CPE mediated effects on cytokine production in the lower intestine could be locally limited to the intestinal epithelium. Therefore, we studied the effects of colonic crypt supernatants obtained from *cpe* ^−/−^ and *cpe* ^+/+^ mice after incubation with forskolin on MODE-K cells, a murine duodenal IEC line. Similar to the supernatants of LPS stimulated colonic biopsies we found decreased levels of NPY and PYY as assessed by ELISA (data not shown). After incubation of MODE-K cells with the respective supernatants in the presence or absence of LPS (see Figure 3A for experimental setup) transcript levels of KC and IL-6 were determined via RT-PCR. No significant differences between the groups were measured for both KC and IL-6 without LPS. However, after addition of LPS the expression levels of KC and IL-6 were elevated. Remarkably, the LPS-dependent inductive potential of *cpe* ^−/−^ supernatants was significantly higher than the respective potential of supernatants obtained from *cpe* ^+/+^ mice (Figure 3B, C). In order to study a potential link between the reduced levels of intestinal neuropeptides, we repeated the experiment in the presence of recombinant NPY and PYY. Notably, co-administration with NPY leads to an approximately 40% reduction of KC expression in MODE-K cells independently of the supernatant's origin (*LPS*: 100%±19.24 (*cpe* ^−/−^) vs. 100%±36.32 (*cpe* ^+/+^); *LPS+NPY*: 63.58%±14.34 (*cpe* ^−/−^) vs. 66.6%±38.8 (*cpe* ^+/+^); *LPS+NPY+PYY*: 50.08%±27.5 (*cpe* ^−/−^) vs. 68.78%±25.76 (*cpe* ^+/+^)) (Figure 3D). Similar, NPY co-treatment also reduced the expression levels of IL-6 (*LPS*: 100%±64.0 (*cpe* ^−/−^) vs. 100%±115.54 (*cpe* ^+/+^); *LPS+NPY*: 69.6%±58 (*cpe* ^−/−^) vs. 62.0%±38.81 (*cpe* ^+/+^); *LPS+NPY+PYY*: 70.48%±58.34 (*cpe* ^−/−^) vs. 79.0%±105.34 (*cpe* ^+/+^)), although not reaching statistical significance (Figure S1F). Finally, treatment with PYY together with NPY did not augment the effect of NPY alone. In conclusion, our data suggest that the lack of neuropeptides resulting from CPE deficiency alters IEC cytokine production.

### Differential chemotactic potential of colonic crypt supernatants

In order to study a possible mechanistic link between the modulation of EEC function by CPE through processing and sorting of gastrointestinal neuropeptides and primary immune cells, which drive intestinal inflammation, we finally tested the effect of supernatants obtained from forskolin-treated crypt cells from *cpe* ^−/−^ and *cpe* ^+/+^ mice on the migratory capacity of BMDM. In relation to supernatants obtained from *cpe* ^+/+^ mice, those from *cpe* ^−/−^ mice exhibited a significantly higher chemotactic effect on wildtype BMDM (261.99%±136,27, p<0,001) as shown by the numbers of migrated BMDM (Figure 3E, F). Interestingly, migration of BMDM isolated from *cpe* ^+/+^ and *cpe* ^−/−^ mice was similar (data not shown), indicating that the chemoattractive supernatants from *cpe* ^−/−^ mice are responsible for the observed effect and not an altered migratory capacity of the respective BMDM.

## Discussion

EEC are crucially involved in the maintenance of intestinal homeostasis, and EEC dysfunction is discussed to play a relevant pathophysiologic role in IBD [11]. With CPE being a possible modulator of EEC function, *cpe* ^−/−^ mice represent a potential model for the investigation of EEC dysfunction and its relevance in intestinal inflammation.

In the present study, we demonstrate a cellular co-localization of CPE with the EEC markers NPY, PYY and CgB in the lower GIT. This result indicates the specific expression of CPE in EEC of the intestinal mucosa. Moreover, our data provide evidence that the intestinal levels of neuropeptides are significantly influenced by CPE. Both analysed neuropeptides, NPY and PYY, are reduced in *cpe* ^−/−^ mice compared to wildtype littermates. According to current knowledge, only precursors of NPY and not PYY are directly processed by CPE in neuroendocrine tissue [33], implicating a more general effect of CPE for EEC function. Here, the known sorting function of CPE for neuropeptides could be a factor of higher relevance than the exopeptidase function on single neuropeptides [34], [35]. Considering that in IBD patients the levels of many different neuropeptides have been found to be altered, we suggest that the *cpe* ^−/−^ model might be a valuable tool to study the complex interplay of EEC derived neuroendocrine factors in the intestines.

In order to investigate the phenotype of *cpe* ^−/−^ mice in the context of intestinal inflammation, we induced chronic DSS colitis as an established murine model of human IBD. The major finding here was a significantly more severe phenotype of colitis in *cpe* ^−/−^ mice as shown by MEICS, DAI and histology, indicating that CPE deficiency modulates the susceptibility of respective mice to experimental colitis.

Moreover, by analyzing intestinal biopsies obtained from DSS treated mice for cytokine expression, we accordingly found significantly increased levels of the proinflammatory cytokines TNF-α, IL-6 and KC as compared to the control group. Surprisingly, we also detected increased KC and IL-6 levels in *cpe* ^−/−^ mice under baseline conditions, suggesting a relevant role of CPE for basal cytokine production. In this regard, several neuropeptides expressed in the intestine have already been shown to affect cytokine levels *in vivo* [28], [36], [37].

The number of primary immune cells in the mucosa, as the main producers of many cytokines [38], was low at baseline conditions and did not significantly differ between both mouse strains (data not shown). This raises the question of alternative sources for the increased IL-6 and KC levels in *cpe* ^−/−^ mice at baseline. In this regard, both IL-6 and KC have been reported to also be expressed by IEC [30]–[32], [39], suggesting that the source of the observed cytokine expression is located in the epithelium and not in primary immune cells, such as lymphocytes and macrophages. In order to test this hypothesis, we used MODE-K cells, a murine intestinal epithelial cell line, and incubated them with supernatants of forskolin-stimulated intestinal crypts isolated either from *cpe* ^−/−^ mice or wildtype littermates. Additionally, cells were treated with LPS, which is constantly present under physiological conditions in the intestine. As the result, MODE-K cells showed significantly increased expression level of KC and IL-6 when stimulated with supernatants of *cpe^−/−^* mice. Interestingly, this effect was reversible by co-administration of recombinant NPY to the supernatants. However, additional administration of PYY did not show a statistical significant effect on KC expression levels.

These data indicate that the elevated cytokine levels observed under baseline conditions in *cpe* ^−/−^ mice might originate rather specifically from IEC, highlighting an important role of CPE and its downstream targets for the immunological environment in the intestinal mucosa, orchestrated by IEC themselves. Moreover, our results highlight the role of NPY as an anti-inflammatory molecule, a property which has been recently demonstrated on myeloid derived primary immune cells [25]. Considering that the cellular processing of NPY is known to be dependent on CPE, our data establish a new link between CPE and NPY as executers of intestinal immune regulation.

In order to study the consequences of altered mucosal cytokine expression in *cpe* ^−/−^ mice, we finally employed a chemotaxis experiment with respective crypt supernatants of *cpe^−/−^* and *cpe* ^+/+^ mice. As the result, bone marrow-derived macrophages showed increased migratory behavior towards the supernatants obtained from *cpe^−/−^* mice, irrespective of BMDM origin from *cpe* ^−/−^ or *cpe* ^+/+^ mice. This might be a further explanation of the observed proinflammatory phenotype of *cpe* ^−/−^ mice upon DSS administration. In this context, primary immune cells invade the intestinal mucosa and have been described to be crucially involved in chronic DSS colitis [40]–[42].

In summary, we demonstrated increased susceptibility of *cpe* ^−/−^ mice against chronic DSS colitis highlighting an important role of CPE for intestinal homeostasis. As a potential mechanism we identified CPE to modulate epithelial cytokine production and thus to be involved in the orchestration of the local immunological environment regulating the migration of primary immune cells. However, more studies are needed to dissect the executers and molecular mechanisms of the CPE mediated effects. Furthermore, future investigation is necessary in order to examine whether the patients with CD who have autoantibodies against EEC or genetic polymorphisms in the Phox2B gene, also show a defective local immune system as demonstrated here in *cpe* ^−/−^ mice. Potentially, these patients might especially benefit from local substitution with GI-neuropeptides or pharmacological modification of EEC function.

## Supporting Information

Figure S1 (A) Determination of expression levels of different neuropetides in colonic punch biopsies by real time RT-PCR at baseline. n = 6 per genotype. (B-E) Immunofluorescence staining of colonic biopsies from *cpe^+/+^* and *cpe^−/−^* mice for CD3, F4/80 and Ly6G (B) and quantification of immune cells in colonic biopsies by counting CD3 (C), F4/80 (D) and Ly6G (E) positive cells per high power field (magnification 40x). 5 random HPF per animal, n = 6 per genotype. (F) IL-6 transcript levels produced in MODE-K cells after incubation with forskolin-stimulated supernatants of of *cpe* ^+/+^ and *cpe* ^−/−^ mice and LPS together with recombinant NPY +/− PYY (1 µM/ml). IL-6 expression levels are expressed in percent of the Mean of *cpe^+/+^*. *p<0.05; ns =  not significant, by t-test.
(JPG)
