# Supplementary figures and images for: Carboxypeptidase E Modulates Intestinal Immune Homeostasis and Protects against Experimental Colitis in Mice
Source: PLoS One. 2014 Jul 22;9(7):e102347. doi: 10.1371/journal.pone.0102347 (PMC4106776; doi:10.1371/journal.pone.0102347)

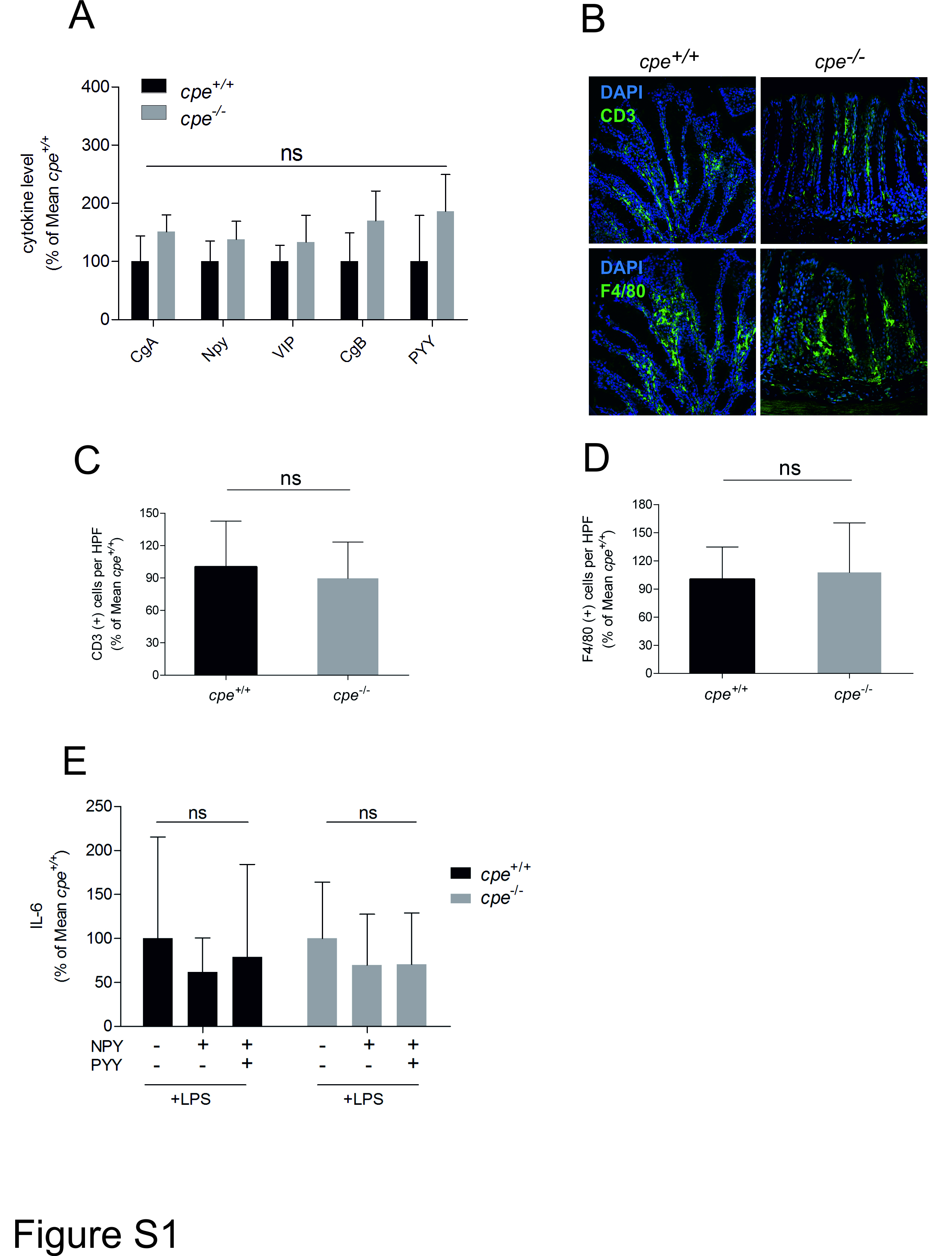

Supplement: Figure S1 — (A) Determination of expression levels of different neuropetides in colonic punch biopsies by real time RT-PCR at baseline. n = 6 per genotype. (B-E) Immunofluorescence staining of colonic biopsies from cpe+/+ and cpe−/− mice for CD3, F4/80 and Ly6G (B) and quantification of immune cells in colonic biopsies by counting CD3 (C), F4/80 (D) and Ly6G (E) positive cells per high power field (magnification 40x). 5 random HPF per animal, n = 6 per genotype. (F) IL-6 transcript levels produced in MODE-K cells after incubation with forskolin-stimulated supernatants of of cpe +/+ and cpe −/− mice and LPS together with recombinant NPY +/− PYY (1 µM/ml). IL-6 expression levels are expressed in percent of the Mean of cpe+/+. *p<0.05; ns = not significant, by t-test. (JPG) [file pone.0102347.s001.jpg]
